# International genomic definition of pneumococcal lineages, to contextualise disease, antibiotic resistance and vaccine impact

**DOI:** 10.1016/j.ebiom.2019.04.021

**Published:** 2019-04-16

**Authors:** Rebecca A. Gladstone, Stephanie W. Lo, John A. Lees, Nicholas J. Croucher, Andries J. van Tonder, Jukka Corander, Andrew J. Page, Pekka Marttinen, Leon J. Bentley, Theresa J. Ochoa, Pak Leung Ho, Mignon du Plessis, Jennifer E. Cornick, Brenda Kwambana-Adams, Rachel Benisty, Susan A. Nzenze, Shabir A. Madhi, Paulina A. Hawkins, Dean B. Everett, Martin Antonio, Ron Dagan, Keith P. Klugman, Anne von Gottberg, Lesley McGee, Robert F. Breiman, Stephen D. Bentley, The Global Pneumococcal Sequencing Consortium

**Affiliations:** a Parasites and microbes, Wellcome Sanger Institute, Hinxton, UK; b New York University School of Medicine, New York, NY, USA; c Faculty of Medicine, School of Public Health, Imperial College London, UK; d Department of Biostatistics, University of Oslo, 0317 Oslo, Norway; e Department of Computer Science, Helsinki Institute for Information Technology HIIT, Espoo, Finland; f Instituto de Medicina Tropical, Universidad Peruana Cayetano Heredia, Lima, Peru; g Department of Microbiology, Carol Yu Centre for Infection, Queen Mary Hospital, The University of Hong Kong, Hong Kong, China; h Centre for Respiratory Diseases and Meningitis, National Institute for Communicable Diseases, Johannesburg, South Africa; i Malawi-Liverpool-Wellcome-Trust Clinical Research Programme, Blantyre, Malawi; j NIHR Global Health Research Unit on Mucosal Pathogens, Division of Infection and Immunity, University College London, London, UK; k WHO Collaborating Centre for New Vaccines Surveillance, Medical Research Council Unit The Gambia at London School of Hygiene & Tropical Medicine, Atlantic Boulevard, Fajara, PO Box 273 Banjul, the Gambia; l The Faculty of Health Sciences, Ben-Gurion University of the Negev, Beer-Sheva, Israel; m Medical Research Council: Respiratory and Meningeal Pathogens Research Unit, University of the Witwatersrand, South Africa; n Department of Science and Technology, National Research Foundation: Vaccine Preventable Diseases, University of the Witwatersrand, South Africa; o Rollins School Public Health, Emory University, USA; p Queens Research Institute, University of Edinburgh, UK; q Division of Microbiology & Immunity, Warwick Medical School, University of Warwick, Coventry, CV4 7AL, UK; r Centers for Disease Control and Prevention, Atlanta, USA; s Emory Global Health Institute, Atlanta, USA

## Abstract

**Background:**

Pneumococcal conjugate vaccines have reduced the incidence of invasive pneumococcal disease, caused by vaccine serotypes, but non-vaccine-serotypes remain a concern. We used whole genome sequencing to study pneumococcal serotype, antibiotic resistance and invasiveness, in the context of genetic background.

**Methods:**

Our dataset of 13,454 genomes, combined with four published genomic datasets, represented Africa (40%), Asia (25%), Europe (19%), North America (12%), and South America (5%). These 20,027 pneumococcal genomes were clustered into lineages using PopPUNK, and named Global Pneumococcal Sequence Clusters (GPSCs). From our dataset, we additionally derived serotype and sequence type, and predicted antibiotic sensitivity. We then measured invasiveness using odds ratios that relating prevalence in invasive pneumococcal disease to carriage.

**Findings:**

The combined collections (*n* = 20,027) were clustered into 621 GPSCs. Thirty-five GPSCs observed in our dataset were represented by >100 isolates, and subsequently classed as dominant-GPSCs. In 22/35 (63%) of dominant-GPSCs both non-vaccine serotypes and vaccine serotypes were observed in the years up until, and including, the first year of pneumococcal conjugate vaccine introduction.

Penicillin and multidrug resistance were higher (*p* < .05) in a subset dominant-GPSCs (14/35, 9/35 respectively), and resistance to an increasing number of antibiotic classes was associated with increased recombination (R^2^ = 0.27 *p* < .0001). In 28/35 dominant-GPSCs, the country of isolation was a significant predictor (*p* < .05) of its antibiogram (mean misclassification error 0.28, SD ± 0.13).

We detected increased invasiveness of six genetic backgrounds, when compared to other genetic backgrounds expressing the same serotype. Up to 1.6-fold changes in invasiveness odds ratio were observed.

**Interpretation:**

We define GPSCs that can be assigned to any pneumococcal genomic dataset, to aid international comparisons. Existing non-vaccine-serotypes in most GPSCs preclude the removal of these lineages by pneumococcal conjugate vaccines; leaving potential for serotype replacement. A subset of GPSCs have increased resistance, and/or serotype-independent invasiveness.

**Research in context**

**Evidence before this study**

We searched PubMed using the terms “*Streptococcus pneumoniae*” OR “pneumococcus” AND “genome sequencing” OR “invasiveness disease potential” AND “genotype” OR “clone”, for papers published in English between Jan 1, 2000, and Aug 21, 2018. Whole genome sequencing of pneumococci has mainly been used for detailed characterisation of strains or lineages. While pneumococcal population structure influences pneumococcal conjugate vaccine impact, only a small number of in-depth descriptions have been performed using species-wide genomic surveys of carriage or disease: Maela, Thailand [3085 isolates], Southampton UK [672 isolates], Massachusetts US [616 isolates], Blantyre, Malawi [585 isolates], Nijmegen, The Netherlands [346 isolates]. These studies all used Bayesian Analysis of Population Structure, which provides robust but dataset specific clustering. Publications over the last 15 years have periodically indicated that genotype influences invasiveness, for some pneumococcal lineages, but this subject has received little attention relative to serotype.

**Added value of this study**

An international genome-based scheme for defining pneumococcal population structure, allowed us to characterise and compare lineages across countries, giving international context to serotype, antibiotic resistance and geographical spread. This study uses GPSC definition of genotype to measure invasiveness, adding to the evidence that genotype can influence invasiveness.

**Implications of all the available evidence**

An international definition of pneumococcal population structure, unifies current and future genomic collections, facilitating comparisons. Increasing knowledge of geographical spread, distribution of antibiotic resistance, existence of non-vaccine-type variants and the invasive contribution of genotypes, provides useful context for assessing PCV impact. The generated genomic data offers a considerable resource, to further investigate the biology behind key themes in global control of pneumococcal disease.

Alt-text: Unlabelled Box

## 1 Background

Pneumococcal conjugate vaccines (PCVs) are highly effective in the prevention of invasive pneumococcal disease, caused by vaccine serotypes [1]. PCVs with seven, 10 or 13 conjugated serotypes are in use in ~150 countries [2]. Further conjugate vaccines are in development including PCV15 (Merck) [3] and a 20-valent formulation (Pfizer) [4], both in phase III clinical trials. Young children are the main carriers of *Streptococcus pneumoniae*, and immunisation of this age group protects them against invasive pneumococcal disease (IPD), caused by vaccine serotypes. Replacement of vaccine serotypes in carriage and disease by non-vaccine serotypes, termed serotype replacement, has offset some of the disease reductions in vaccinated and unvaccinated age groups [5,6]. The pneumococcal capsular polysaccharides are used to classify the pneumococcus into ~100 serotypes based on antibody binding to specific epitopes. Serotype is considered the primary pneumococcal virulence determinant [7].

Pneumococcal population structure can be characterised using multi-locus sequence typing (MLST), that determines the sequence variation in seven housekeeping genes. At least one MLST gene (*ddl*) has been linked to a known recombination hotspot in pneumococci [8]. MLST is limited in its ability to infer relationships between all strains [9], as shared ancestry can be masked by recombination and variation that has accumulated over longer timescales. Whole genome sequencing has increased resolution, allowing relationships between strains across the species to be established. Public health bodies are now taking steps to integrate pneumococcal whole genome sequencing into routine microbiology. Public Health England and the Centers for Disease Control and Prevention, have published methods using pneumococcal whole genome sequencing for determining serotype, and predicting antibiotic susceptibility [10,11].

Multiple studies have used pneumococcal genomics to investigate PCV impact [[12], [13], [14], [15]]. They often cluster the population into groups using genomic variation that reflects a shared evolutionary history. To date, these genomic definitions of lineages have been dataset-specific, unlike MLST, hindering their use when comparing studies. The Global Pneumococcal Sequencing project (GPS, http://www.pneumogen.net/gps/), aims to provide an international understanding of pneumococcal population structure and PCV impact. It includes pneumococcal collections from invasive disease, and asymptomatic carriage. Multiple low- and middle-income countries are represented, and where possible, samples collected before and after PCV introductions. We aimed to use genome-wide variation to capture signals of shared descent, and define Global Pneumococcal Sequence Clusters (GPSCs). We used the GPSCs to provide further context on the distribution of serotypes, antibiotic resistance and invasiveness across pneumococcal lineages, which can aid assessments of PCV-impact [16,17].

## 2 Methods

### 2.1 Study design

We included 13,454 pneumococcal genomes available from the ongoing GPS project by June 2017. Investigators from each country provided epidemiological information including clinical manifestation, host age group, isolation year and sample source (Supplementary T1). IPD isolates were from a normally sterile site, while carriage isolates were from nasopharyngeal or nasal swabs. Participating laboratories performed antibiotic susceptibility testing where facilities allowed. We interpreted the susceptibility data as SIR (susceptible, intermediate, resistant) using Clinical Laboratory Standard Institute (CLSI) M100-ED28:2018. We applied the meningitis threshold for penicillin on all isolates, to allow assessment and comparison of penicillin resistance between GPSCs. Phenotypic antibiotic susceptibility data were available for <50% of isolates. This available data could be used to assess the sensitivity and specificity of genotypic prediction, and the validity of generating new, genome-derived, susceptibility data for several countries. The pre-PCV period was defined as the years when no conjugate vaccine was used and the year of first PCV introduction in each country.

### 2.2 Whole genome analysis

The following whole genome analyses are expanded in Supplementary Methods. Briefly, isolates were Illumina sequenced and raw data, assembled [18] and deposited in the European Nucleotide Archive (Supplementary T1). We derived MLST sequences types (STs) and serotype from the genome [19,20]. We further screened for the presence of known resistance conferring genes and mutations – for penicillin, tetracycline, erythromycin, chloramphenicol, co-trimoxazole – in the CDC pneumococcal typing pipeline database [11,21,22]. References to serotype and resistance throughout are from genomic inference. Multidrug resistance was defined as predicted resistance to ≥ 3 antibiotic classes.

To define GPSCs and improve global representation, the GPS dataset (*n* = 13,454) was supplemented with published datasets from the Netherlands (*n* = 2803), Thailand (*n* = 2663), USA (*n* = 616) and UK (*n* = 491) (Supplementary T2). We used PopPUNK to group isolates from this combined dataset (*n* = 20027) into lineages, which clusters them using core and accessory distances (Fig. S1) [23]. We coined these lineages Global Pneumococcal Sequence Clusters (GPSCs), and created a reference database – available at https://www.pneumogen.net/gps/assigningGPSCs.html – that can be used with popPUNK to assign the GPSCs to new data. HierBAPS was used as a clustering comparator [24]. It was run on a SNP alignment generated by mapping GPS isolates to ATCC 700669. Recombination was quantified among core genes using FastGEAR on a representative set of STs (Supplementary T3). Recombination within each dominant-GPSC was quantified using Gubbins after mapping to a GPSC specific reference (Supplementary T4) [25,26]. Pairwise SNP distances were calculated for a core gene alignment of the GPS dataset generated *via* Roary [27], and for recombination-free alignments per dominant-GPSC, using the Pairsnp-r R package.

### 2.3 Statistical analysis

Estimates of the number of GPSCs in the true population were modelled using the R Vegan package [28]. Simpson's Diversity index 1-D (SDI) reports no diversity (zero) to unlimited diversity (one). We tested the predictive value of dominant-GPSCs, for antibiogram or serotype. We then tested the predictive value of country, for antibiogram or serotype, within each dominant-GPSC. We compared multinomial logistic regressions to null models using a likelihood ratio test. Input data was restricted to the un-perturbed pre-PCV population. Corrections for multiple testing (*n* ≥10) used the Benjamini-Hochberg false discovery rate of 5%. Pneumococcal heritability (*h*^2^) of invasiveness was calculated with a regression of all genomic variation using Pyseer [29] on South African isolates, from children <7 years of age in which variation in invasiveness is limited (Supplementary T5) [30]. The explanatory value of serotype for invasiveness was measured using Nagelkerke's pseudo-R^2^ [31]. Quantification of invasiveness was performed using odds-ratios (OR) for invasive disease where prevalence in invasive disease was related to their prevalence in carriage. We performed an OR meta-analysis of data from South Africa (national IPD *n* = 625, carriage; Agincourt *n* = 798, Soweto *n* = 291) and USA (national IPD *n* = 456, carriage Massachusetts *n* = 345), using individuals <7 years old (Supplementary T6). The Cochran's Q-test was used to detect heterogeneity by country within estimated ORs. The (log) odds ratio for invasive disease used Peto's method where *a* is the number of invasive isolates of X, where X denotes a particular serotype, genotype or serotype-genotype, *b* is the number of carriage isolates of X, *c* is the number of non-X invasive isolates, and *d* is the number of non-X carriage isolates, in line with previous work by Brueggemann *et al* [7]. Measuring differences in the proportion of IPD cases to carriage were performed between the pairs of most and least invasive genotypes, using the OR dataset from the country (USA or South Africa) they were predominantly observed. These statistical analyses are expanded in Supplementary Methods.

## 3 Findings

Our GPS dataset included 13,454 isolates representing 30 countries, and 5 continents: Africa (13 countries, 59% of collection), Asia (8, 18%), South America (2, 8%), Europe (4, 3%) and North America (3, 12%). GPS key countries (*n* > 1000) included South Africa (*n* = 4615, 63% IPD), The Gambia (*n* = 1647, 24% IPD), Malawi (*n* = 1304, 43% IPD), Israel (*n* = 1143, 100% IPD) and USA (*n* = 1584, 100% IPD). Sixty-four percent of the collection were isolated from IPD, 96% of the collection were isolated between 2000 and 2017 and 74% were from children aged ≤5 years old (Table 1, Fig. S2).

**Table 1:** Clinical characteristics, age group, sex, clinical manifest and sample source, PCV period.

Category	≤2	3–5	6–15	16–24	25–44	45–65	>65	Total
Total	7179	2790	1061	398	982	602	442	13,454
**Sex**								
F	2863	972	440	214	502	173	89	5253
M	3288	1067	486	150	343	209	117	5660
Missing	1028	751	135	34	137	220	236	2541
**Manifest**								
Carriage	2841	935	646	223	173	25	6	4849
Disease	4338	1855	415	175	809	577	436	8605
**Source**								
Blood	2998	1234	256	93	471	404	332	5788
CSF	1070	444	135	67	290	132	55	2193
Nasopharynx	2841	934	646	223	173	25	6	4848
Other disease (non-invasive)	256	173	24	15	45	38	46	597
Missing	14	5	0	0	3	3	3	28
**PCV period**								
Pre-PCV	3925	1439	671	250	418	230	177	7110
Post-PCV7	1355	547	153	52	211	143	113	2574
Post-PCV10	131	44	13	7	22	44	12	273
Post-PCV13	1592	671	210	86	314	177	131	3181
No universal introduction of PCV	176	89	14	3	17	8	9	316

Isolates were classified by PCV use in the country and year of their isolation. The pre-PCV period was defined as the years when no conjugate vaccine was used and the year of first PCV introduction, the post-PCV7 period from the second year of PCV7 introduction through to the first year of PCV10 introduction, the post-PCV10 period from the second year of PCV10 introduction through to the first year of PCV13 introduction and the post-PCV13 period from the second year of PCV13 introduction through to the last collection year. No universal introduction of PCV denotes years in which a nationwide immunisation policy did not exist.

Genome-wide variation in our dataset combined with published collections (total *n* = 20,027) clustered isolates into 621 GPSCs (Fig. S3). Our 621 GPSCs represent over 61% of the 1012.7 GPSCs (SE ±76) estimated to be in the true population. However, most GPSCs (407 of 621, 66%) were rare lineages with <10 isolates, together representing 1043 of 20,027 (5%) of the combined collection. Within the GPS dataset, we observed 538 GPSCs. 35 GPSCs had >100 isolates in the GPS dataset and were classified as dominant-GPSCs. Together they represented 8356 of 13,454 (62%) of the GPS dataset, and 5978 of 8605 (69%) of the GPS dataset disease isolates (Fig. S4). Sampling multiple countries detected significantly more GPSCs (*p* < .0001) than equivalent sampling from a single location (Fig. S5).

**Fig. 1:**
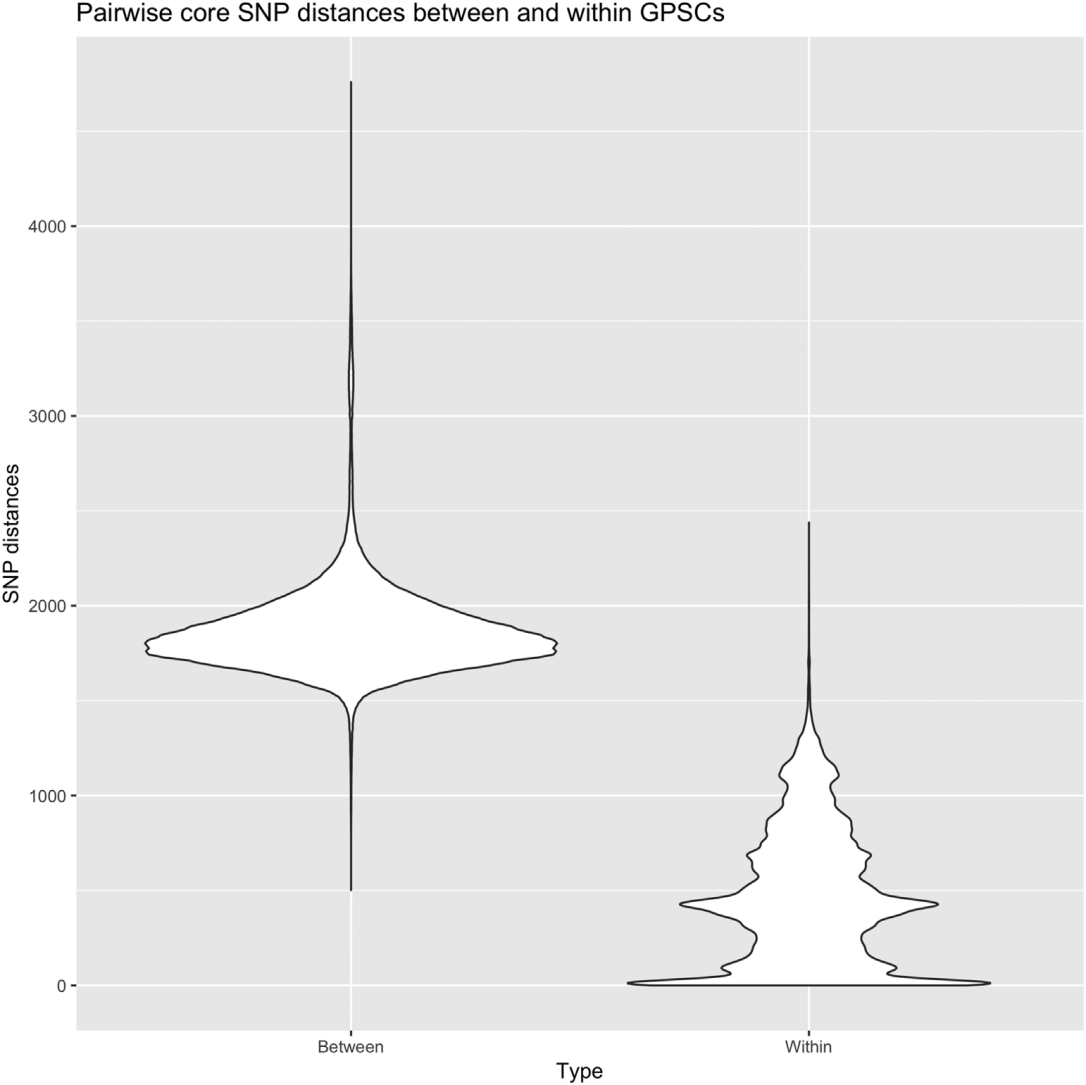
Pairwise core SNP distances between and within GPSCs. Pairwise SNP distances, from a core alignment generated using Roary, between isolates in different GPSCs (left) are generally greater than pairwise SNP distances for isolates within the same GPSC (Right).

**Fig. 2:**
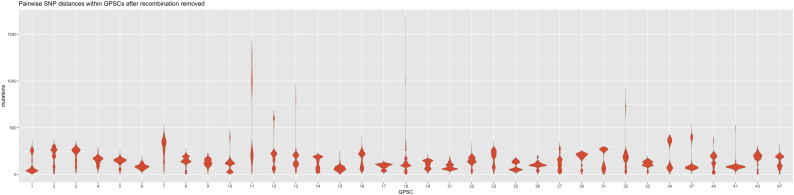
Pairwise SNP distances within dominant-GPSCs. Violin plots of pairwise SNP distances within the 35 dominant-GPSCs after recombination removed from an alignment from mapping to an internal reference for each GPSC are largely comparable.

We defined MLST Clonal Complexes (CC) as STs with single locus variant (SLV) differences, within the GPS dataset. GPSCs often encompassed related CCs, with a mean number of 2.6 CCs per dominant-GPSC (SD ±1.5, excluding singleton STs). GPSCs identify a shared history not captured by CC designations. CC217, CC615, CC3581 and 2 singleton STs were clustered into GPSC2, a grouping of CCs which is widely recognised as a clonal lineage that expresses serotype 1. CCs captured phylogenetic sub-structure well in dominant-GPSCs with more than 1 CC (*n* = 25, mean Consistency Index 0.9864 SD ± 0.04, mean Retention Index 0.9942 SD ± 0.02). Our clustering additionally revealed shared descent of CC53, CC1012, CC62 and CC100 within GPSC3, that shared 0–5 MLST alleles. HierBAPS supported the clustering of 28/35 (80%) dominant-GPSCs, including GPSC3. The species-wide, core genome, pairwise SNP distances between GPSCs and within GPSCs, were predominantly non-overlapping (Fig. 1). The mean pairwise SNP distances from recombination free alignments, were broadly comparable between dominant-GPSCs, though eight GPSCs had SNPs distances of >500 for a subset of their isolates (Fig. 2). HierBAPS supported half of those clusters with >500 SNP distances, but GPSC18, GPSC23, GPSC37 and GPSC41 were split into two sub-clusters. Conversely GPSC1, the clonal serotype one lineage GPSC2 and GPSC16 were split by HierBAPS into two sub-clusters even when the maximum SNP distances were <500.

MLST genes g*ki*, *gdh*, *recP*, *spi* and *ddl* ranked in the top 6–22% of the 1193 core genes, for recombination frequency (Supplementary T7). Disruption of vertical inheritance may result in isolates that are missed by CC: within the dominant-GPSCs 370 of 8356 isolates (4.4%), belonging to 165 STs, were not assigned to a CC. Conversely recombination can result in convergent MLST profiles in disparate isolates, and CC designation using large collections are more vulnerable to spurious connections. Sixteen CCs spanned multiple related GPSCs and 24 isolates were assigned to 6 CCs highly discordant with their core genome phylogenetic relationship (Fig. S6).

**Fig. 3:**
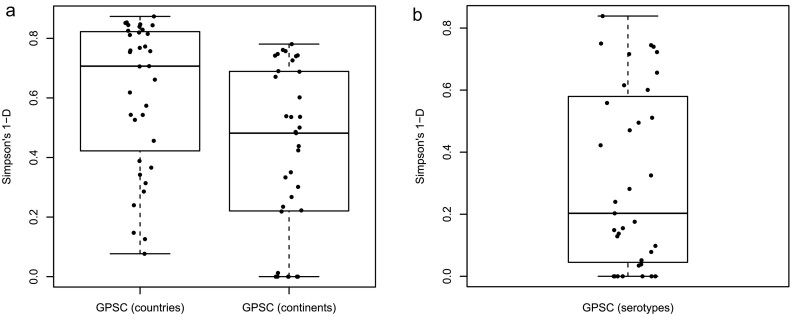
Geographical and serotype diversity within Global Pneumococcal Sequence Clusters (GPSCs). Boxplots of minimum, first quartile, median, third quartile, and maximum values. (A) geographical diversity of dominant-GPSCs by country or continent, or (B) serotype diversity of dominant-GPSCs. Each point represents a dominant-GPSC. Only the unperturbed pre-PCV isolates was used to capture serotype diversity per GPSC. Diversity was measured using Simpsons 1-D that considers the number of locations/serotypes present, as well as the relative abundance of each location/serotype. Zero denotes no diversity and 1 denotes unlimited diversity.

Geographical diversity varied considerably per GPSC (Fig. 3A), though all dominant-GPSCs were observed in more than one country. Eight of 35 (23%) had high geographical diversity (SDI >0.70) representing even distribution across 5 continents (Supplementary T8). Conversely, seven of 35 (20%) dominant-GPSCs were observed only in Africa.

Genomic inference of serotype was reliable with 97% (95% CI 97.15–97.75%) serotype co-concordance with available phenotypic data (*n* = 10,466, Supplementary T9). Of 35 dominant-GPSCs, 30 (86%) were observed with more than one serotype. Dominant-GPSC was a significant predictor of serotype with a mean misclassification error of 0.18, although serotype diversity within dominant-GPSCs varied considerably (*p* < .0001), Fig. 3B). Country was a significant predictor of serotype within 18 of 35 dominant-GPSCs (51%) with a mean misclassification error of 0.19 (SD ± 0.14, *p* < .05). For example, in GPSC3, serotype 8 was expressed in 113 of 148 (76%) IPD isolates from South Africa and all were CC53, conversely, serotype 33F was expressed in 49 of 87 IPD isolates from the USA and 44 of 49 (90%) were CC100/ST2705.

**Fig. 4:**
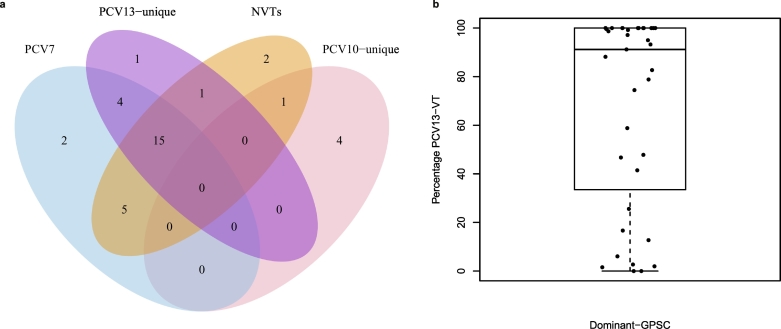
PCV composition of dominant Global Pneumococcal Sequence Clusters (GPSCs). (A) Venn diagram of the number of dominant-GPSCs (*n* = 35) in which combinations of PCV7 VTs, PCV10/13 unique VTs and NVTs were observed in the same GPSC pre-PCV. Over one-third (15/35) of the dominant-GPSCs expressed both PCV7 (blue), PCV13-unique (purple) and NVT (orange) pre-PCV, shown in the overlap. Whilst 24/35 dominant-GPSCs had at least one isolate expressing an NVT pre-PCV (orange area). (B) PCV13 VT contribution to GPSCs pre-PCV. Each point represents a dominant-GPSC (*n* = 35) and the percentage of its pre-PCV isolates expressing PCV13-VTs, with boxplot of minimum, first quartile, median, third quartile, and maximum values. (For interpretation of the references to colour in this figure legend, the reader is referred to the web version of this article.)

Two of the 35 dominant-GPSC (6%, GPSC14, GPSC37) included isolates exclusively expressing PCV7-serotypes pre-PCV and therefore were completely covered by PCV7. However, GPSC14 and 37 accounted for only 169 of 4221 (4%) of pre-PCV disease isolates. The number of dominant-GPSCs expressing only VTs pre-PCV, increased to 6 GPSCs for PCV10 (885 of 4221 [21%] of pre-PCV IPD isolates), and 11 GPSCs for PCV13 (1234 of 4221 [29% pre-PCV IPD]; Fig. 4A). The experimental 15-valent vaccine offered no additional dominant-GPSCs expressing only VTs. The putative 20-valent formulation meant that a further four dominant-GPSCs were expressing only VTs pre-PCV (1592 of [37%] of pre-PCV IPD isolates).

Of the 35 dominant-GPSCs, 22 (63%) expressed non-vaccine serotypes (NVT) alongside PCV13 serotypes prior to PCV introductions. Though there were wide variations in the ratio of VT:NVT expressed (Fig. 4B). Of the 22 GPSCs, 12 (55%) predominantly expressed VTs. NVT-variants were not observed in all locations, in South Africa, 17 of 34 (50%) GPSCs with 10 or more isolates pre-PCV (*n* = 1534), expressed both PCV13 and NVTs pre-PCV. There were two dominant and 60 intermediate GPSCs expressing only NVTs, respectively they accounted for 20 of 4221 (0.5%) and 208 of 4221 (5%) of IPD isolates pre-PCV.

**Fig. 5:**
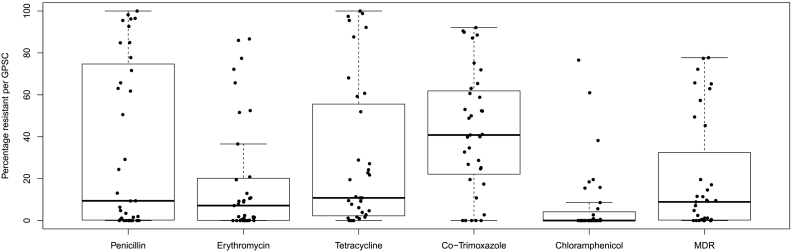
Antibiotic resistance in the dominant Global Pneumococcal Sequence Clusters (GPSCs). Boxplots of minimum, first quartile, median, third quartile, and maximum percentage of pneumococcal isolates with antibiotic resistance to five common classes. Each dot represents a dominant-GPSC (*n* = 35). Only the unperturbed pre-PCV isolates was used to capture the distribution of resistance to each class across the dominant-GPSCs.

The positive predictive values for genetic determinants of resistance to penicillin, tetracycline, erythromycin, chloramphenicol, and co-trimoxazole were all >90% (95%CI 90–98.7%, Supplementary T10). Resistance to at least one antibiotic class was predicted for 8241 of 13,454 isolates in the GPS dataset (61%; Fig. S7). The percentage of isolates predicted to be resistant per class was not uniform across dominant-GPSCs (Fig. 5). The predicted resistance profile of an isolate could be predicted by which dominant-GPSC it belonged to half of the time, (*p* < .0001, misclassification error 0.49). Generally, higher recombination ratios (rho/theta, r/m), were associated with a higher mean number of classes of predicted antibiotic resistance (rho/theta R^2^ = 0.27, *p* < .0001, r/m R^2^ = 0.22, *p* < .0001, Supplementary T8 and T11). GPSC1 had an above average r/m (8.3) and rho/theta (0.14) and a predominant predicted MDR antibiogram of penicillin, co-trimoxazole, erythromycin and tetracycline resistance, but susceptibility to chloramphenicol, occurring in 388 of 504 isolates (77%). Although this MDR antibiogram was the most common pattern in 17 of 19 countries represented in GPSC1, country was a predictor of the antibiogram for 28 of the 35 dominant-GPSCs (80%; *p* < .05, mean misclassification error 0.28, SD ± 0.13). Predicted penicillin resistant isolates accounted for a higher proportion of isolates within 14/35 dominant-GPSCs than expected given proportion of predicted penicillin resistance in the rest of the GPS dataset (*p* < .05, 63–100%, Supplementary T12). Predicted multidrug resistant isolates, accounted for higher proportion of isolates within 9/35 dominant-GPSCs than expected (*p* < .05, 45–77%). Eight of these were GPSCs also found to have a higher proportion of isolates penicillin resistant isolates (Supplementary T13). Prior to PCV introductions, penicillin resistance was predicted to occurred in 2133 of 4975 (43%) of the isolates expressing PCV13 VTs and in only 256 of 2135 (12%) of the NVT expressing strains (*p* < .0001).

In 9 of the 22 (40%) GPSCs expressing both VT and NVT, the NVT component had a significantly lower proportion of predicted resistant isolates than their VT counterparts (Supplementary T14). Seven intermediate GPSCs expressing only NVTs had >90% of isolates predicted resistant to penicillin (GPSC55, 89 of 90, [99%]; GPSC59, 37/37 of [100%]; GPSC81, 35 of 38 [92%]; GPSC132, 17 of 17, 100%]; GPSC136, 19 of 21 [90%]; GPSC168, 15 of 15 [100%]; GPSC200, 11 of 11 [100%]).

In the South African heritability dataset, serotype explained a third of the strain variation in clinical manifestation (carriage or disease, pseudo-R^2^ 0.32). Total pneumococcal genetic variation (including the *cps* locus which encodes the CPS) was a better explanation (*h*^2^ 0.57), explaining over half of the variation in clinical manifestation, leaving some invasiveness explained by genes outside the *cps* locus.

The 95% CI, for invasiveness ORs did not overlap between at least one pair of genotypes (GPSC *n* = 96, ST *n* = 112) within serotypes 6A, 14, 16F, 19F, 23B and 23F (Table 2, Fig. S8). Only within GPSC14 was a genotype with increased invasiveness found to be significant at the ST level but not the GPSC level, as ST6279 and ST2059, both found within GPSC14, significantly differed in invasiveness (Table 2, Supplementary T15 and T16). We detected significant heterogeneity in invasiveness for serotype 38-GPSC38/ST393 (Q = 3.877[df 1], *p* < .05), between South Africa (OR 0.67, 95%CI 0.24–1.88) and the USA (OR 6.83, 95%CI 0.86–54.20) in the meta-analysis estimate of the OR. Despite the small sample numbers, the two countries significantly differed in the proportion of serotype 38-GPSC38 from IPD (*p* = .008). A conservative comparison using the upper CI of the least invasive genotype and the lower CI of the most invasive genotype had 1.05 to 1.6-fold changes in OR (Table 2). The influence of genotype on invasiveness can be of a similar magnitude to some serotypes. For example a 1.6-fold change was observed between the upper CI of the less invasive serotype 35A (0.12[0.016–0.938], *p* = .043) and the lower CI of the more invasive serotype 18C (3.237[1.514–6.921], *p* = .0024) determined in this dataset (Supplementary T17).

**Table 2:** Pneumococcal invasiveness for pairs of genotypes that significantly differed within a serotype.

Serotype	Least invasive (predominant CC)	OR [95% CI]	Most invasive (predominant CC)	OR [95% CI]	Conservative OR fold change	Fisher's *p*-value (country)
6A	GPSC5 (CC172)	0.34 [0.12–1.01]	GPSC41 (CC1094)	2.96 [1.61–5.45]	1.6	0.0004 (ZA)
14	GPSC9 (CC63)	0.62 [0.22–1.73]	GPSC18 (CC15)	12.45 [2.82–54.98]	1.6	0.0005 (ZA)
16F	GPSC33 (CC4088)	0.14 [0.06–0.36]	GPSC46 (CC30)	2.62 [0.44–15.73]	1.2	0.0099 (ZA)
19F	GPSC21 (CC347)	0.32 [0.19–0.54]	GPSC1 (CC320)	1.49 [0.78–2.88]	1.4	0.0006 (ZA)
23B	GPSC7 (CC439)	0.14 [0.05–0.42]	GPSC5 (CC172)	3.81 [0.44–32.79]	1.05	0.005 (USA)
23F	GPSC14 ST6279 (CC6279)	0.81 [0.43–1.54]	GPSC14 ST2059 (CC6279)	5.31 [1.71–16.54]	1.1	0.004 (ZA)

GPSC (Global Pneumococcal Sequence Cluster) OR (Odds ratio), ST (Sequence Type), CC (Clonal Complex), ZA (South Africa), CI (confidence interval), The conservative fold change in OR was calculated by dividing the lower CI of the most invasive genotype by the upper CI of the least invasive genotype, within each serotype.

## 4 Discussion

We present the distribution of key themes in pneumococcal disease control, such as serotype, antibiotic resistance and invasiveness, in a large international collection. We used genome-wide variation to define Global Pneumococcal Sequence Clusters (GPSCs), to produce a dataset independent genomic definition of lineages. Increasing knowledge of the spread of traits across the pneumococcal population and geographical regions, gives greater context for assessing the impact of PCV introduction.

At an international level, we have shown that pneumococcal non-vaccine serotypes exist alongside vaccine serotypes, within dominant GPSCs that account for the majority of the pneumococcal population. The existence of non-vaccine serotype variants negates reliance on contemporaneous capsule switch events for “vaccine escape” of a GPSC. Given that the pneumococcus has multiple lineages that are globally disseminated there is potential for non-vaccine types established in one geographical region to spread globally, or be present but undetected in other countries [32]. Indeed, previous carriage studies have observed the importation of lineages not previously observed in that location, and further estimated that the influx of new lineages would result in a 50% population turnover after 13 years [16,33].

We showed that antibiotic resistance was enriched in a subset of GPSCs, many of which were dominant and globally disseminated. We observed that both GPSC, and country within GPSCs were significant predictors of the antibiotic resistance pattern of an isolate. GPSCs with an increased propensity for resistance, whether associated with increased capacity for recombination, duration of carriage [34], can spread to other locations. Loss of resistance, in the absence of selection, has been reported for lineages in countries with lower antibiotic prescription rates; multiple independent losses of resistance to chloramphenicol, tetracycline and erythromycin were observed for Pneumococcal Molecular Epidemiology Network (PMEN)2 in Iceland [35]. However, over a decade after a reduction in antibiotic consumption, the majority of Icelandic PMEN2 remained resistant, albeit at a reduced prevalence, and as such lineages remain a risk to high usage settings. Antibiotic resistance is lower in non-vaccine serotypes, but this prevalence varies substantially by GPSC. Some notable GPSCs expressing only non-vaccine serotypes do have high levels of penicillin resistance, and within GPSCs that express both non-vaccine and vaccine serotypes, the non-vaccine serotypes occasionally have similar or higher resistance profiles to their vaccine serotype counterparts.

Preservation of gene frequencies in the population through negative frequency-dependent selection has been shown to exist in pneumococci, and can be used to predict serotype replacement in carriage [16,36]. This suggests that the gene content of a GPSCs influences whether it will undergo replacement or expansion after vaccine perturbation. Only genomic data combined with a robust clustering method has the power to model such complex dynamics. Non-vaccine and vaccine serotypes within the same GPSC, will share similar gene complements and ecological phenotypes, including resistance. Non-vaccine variants may therefore have increased potential to replace their vaccine-type counterparts compared to other GPSCs expressing non-vaccine serotypes [13,37]. The GPSCs involved in any replacement will determine the extent to which reductions, not only in total disease, but in antibiotic resistance, could be partially offset by non-vaccine serotypes. This has been observed with pneumococcal serotype replacement after routine use of the 7-valent conjugate vaccine by multidrug resistant 19A in the US within GPSC1(CC320), which slowed reductions in total disease, antibiotic resistance, and subsequently, the cost effectiveness of PCV7 [38].

Serotype is a potent virulence determinant, however other virulence factors exist in the genome outside of the *cps* locus. Genotypes have previously been implicated in invasiveness in a number of small studies using MLST/pulsed-field gel electrophoresis definitions, some of which are complicated by age-related differences in invasiveness [30,[39], [40], [41]]. With a substantial collection of pneumococcal genomes, we have used heritability analysis and difference in invasiveness ORs to demonstrate that genome variation beyond serotype contributes to invasiveness in children <7 years old. Measurable differences in invasiveness ORs between individual GPSCs and STs were comparable to a change in serotype. Stratification of serotype by genotype (GPSC or ST) significantly impacts sample size and subsequent power to detect subtle influences of genotype. Many serotypes were not represented by multiple genotypes in our dataset preventing the contribution of those GPSCs being fully assessed. The generation of further temporally matched and genotyped collections from carriage and disease would increase power and allow further investigation of our findings. Comparing national surveillance with local carriage has the potential to introduce bias, but with sufficient sampling acts as a convenient proxy for the national population. Despite these limitations, differences in invasiveness within serotypes was observed in nearly a quarter (23%, 6/26) of the serotypes tested. Genetic determinants of invasiveness need not be uniform across a genotype; pneumococci within a given genotype will differ slightly in gene content and sequence. This fits with our observations of differences between STs within 23F GPSC14/CC6279, heterogeneity within GPSC38 between countries, and previous work by others showing differing invasiveness of PFGE clones within CC138 [41]. Our work independently observes the increased invasiveness of serotype 14 in a GPSC18/CC15 background compared to at least one other genotype, as previously reported, and we highlight differential invasiveness for genotypes within 6 serotypes in total [41].

There is substantial sequence variation in the *cps* locus within serotypes to acknowledge [42]. Such variation could represent undetected differences in capsular structure, which could in turn influence invasiveness. This was the case for serotype 6C before it was discriminated from 6A, though conversely, divergent genotypes of serotype 6B still result in the same polysaccharide [42,43]. The *cps* loci within serotypes in the GPS collection have been investigated [44]; of the serotypes implicated as differing in invasiveness, only 16F in GPSC33 has an atypical *cps* locus, which may explain why we observed it was less invasive than GPSC46, which has the typical 16F *cps* genotype. After ruling out the influence of novel serotypes, identification of the genetic variation driving the observed invasiveness of GPSCs could offer alternative vaccine targets associated with severe disease. Genome wide association studies (GWAS) have been used by others to identify candidate genetic variation associated with different manifestations of disease [31,45,46].

While MLST is limited in resolution to infer strain relatedness, it is an internationally reproducible scheme. To date an international scheme has not existed for clustering pneumococcal isolates using whole genome data, and alternative methods for clustering genomic collections of this scale are scarce. Furthermore, current methods lack the ability to place a strain in an existing framework, consequently clustering an additional strain would require re-running an entire collection without a reproducible clustering nomenclature. Our definition of the pneumococcal lineages (GPSCs) on an internationally sampled population, can be used to assign GPSCs to any collection of pneumococcal genomes using our database of GPSC reference genomes and PopPUNK [23]. The GPSC database can be updated when novel GPSCs are assigned in future collections, enabling stable international comparisons of pneumococcal population structure [15]. The GPSCs are broadly back-compatible with MLST as the vast majority of STs were found exclusively within a GPSC. To that end, we provide a ST-GPSC conversion table with noted exceptions, to facilitate cross referencing of non-genomic datasets (Supplementary T18). We have used these GPSC designations, genome-derived serotype and antibiotic resistance to facilitate an in depth assessment of the lineages causing invasive disease in young children in the post-PCV13 era [17], and to explore the mechanisms driving the progression of serotype replacement.

Understanding the underlying genetic variation and characteristics of GPSCs that influence resistance, invasiveness and pneumococcal population dynamics in a global context is highly informative. Such information can be used for modelling vaccine replacement, predicting vaccine impact and rational vaccine design. Our high-resolution genomic approach for defining pneumococcal lineages across different collections, in a manner that reflects pneumococcal biology, increases the evidence required to build a global strategy for continued control of pneumococcal disease.

## Appendix A Supplementary data

Supplementary T1-T21 Image 1

GPS consortium members Image 2

Supplementary methods Image 3

Supplementary Figures Image 4
