# Supplementary material for: International genomic definition of pneumococcal lineages, to contextualise disease, antibiotic resistance and vaccine impact
Source: eBioMedicine. 2019 Apr 16;43:338–46. doi: 10.1016/j.ebiom.2019.04.021 (PMC6557916; doi:10.1016/j.ebiom.2019.04.021)
Supplement: Supplementary file 3 — Supplementary methods [file mmc3.docx]

Supplementary whole genome analysis

DNA extracted from single colonies were sequenced on Illumina HiSeq or 10X platforms with Truseq chemistry and >=100bp paired-end reads. Assembly and annotation were performed as previously described, raw data are deposited in the ENA (Supplementary T1)[[18]](https://paperpile.com/c/UToEGL/TPMF). STs were assigned using mlst_check [[19]](https://paperpile.com/c/UToEGL/cp3X). Serotypes were inferred using PneumoCaT and SeroBA -full discrimination was performed by inspecting the *cps* locus if required[[10,47]](https://paperpile.com/c/UToEGL/8ukA2+iAHS). Penicillin binding protein alleles *pbp1A*, *pbp2B* and *pbp2X* were used to predict penicillin MIC mg/l, with CLSI M100-ED28:2018 interpretations. We used the meningitis threshold for penicillin for all isolates, allowing assessment and comparison of penicillin resistance between GPSCs. Etests were interpreted using CLSI broth dilution break points. Genomes were screened for the presence of resistance conferring genes tetracycline (*tet*), erythromycin (*erm* or *mef*), chloramphenicol (*cat*) and resistance conferring mutations for co-trimoxazole (*fol*A, *fol*P) in the CDC pneumococcal typing pipeline database[[11,21,22]](https://paperpile.com/c/UToEGL/g8g4+q6Ak+yxVs). An additional screen for a tetS/M mosaic detected in GPSC10 used ARIBA and tetS/M sequence AY534326 [[48]](https://paperpile.com/c/UToEGL/MlPTA). References to serotype and resistance throughout are from genomic inference, with a correction for PBP 23--0--158 where the phenotype did not support the predicted penicillin MIC of 0.12 (62/63 MIC <0.06). Multidrug resistance was defined as predicted resistance to >=3 antibacterial classes.

To define GPSCs and improve global representation, the GPS dataset n=13,454 was supplemented by published datasets from the Netherlands n=2,803, Thailand n=2,663, US n=616 and UK n=491 (Supplementary T2). PopPUNK was used to assign these isolates n=20,027 to GPSCs. PopPUNK clustered using core and accessory distances, calculated by shared DNA k-mers with lengths between 13 and 29 bases and optimising the decision boundary for isolates clustering in the same or different GPSCs (Figure S1)[[23]](https://paperpile.com/c/UToEGL/9eVBn). We initially used the PopPUNK default Gaussian mixture model with three components, which was then used in the fit refinement mode to optimise the network clustering score. We coined these lineages Global Pneumococcal Sequence Clusters (GPSCs) and created a reference database available at https://www.pneumogen.net/gps/assigningGPSCs.html that can be used with popPUNK to assign the GPSCs to new data. Isolates were assigned to clonal complexes (CC) using the single locus variant (SLV) threshold on the GPS dataset (Supplementary T19). One isolate representing each GPSC (Supplementary T20) was mapped against the ATCC 700669 23F reference [[49]](https://paperpile.com/c/UToEGL/TPk3f), the resulting alignment was reduced to variable sites and FastTree used to produce a phylogeny to visualize the congruence of CCs andGPSCs [[50,51]](https://paperpile.com/c/UToEGL/DyHIu+NLFcn). HierBAPS clustering was run on a SNP alignment from mapping n=11,378 isolates from the GPS dataset to ATCC 700669 (Supplementary T21) [[24]](https://paperpile.com/c/UToEGL/uFG2).

Roary was used to define the core genes without splitting paralogs on the GPS dataset, and a subsample of genomes (n=2463) representing each of the observed STs (Supplementary T3). The ST representative core gene alignment was screened for recombination by FastGEAR to quantify recombination in the MLST genes [[25,27]](https://paperpile.com/c/UToEGL/pBo3O+IrI0A). We mapped dominant-GPSCs isolates to an appropriate reference (Supplementary T4). Where a publicly available whole genome reference was of an ST represented within an GPSC it was used as the reference for analysis (n=10). An additional four references were created using PacBio data from GPS isolates. PacBio reads were assembled with HGAP v3, short contigs already represented in the chromosome were removed [[52]](https://paperpile.com/c/UToEGL/DyftH). The resultant assembly was polished with the illumina reads using Pilon and circularized with Circlator [[53,54]](https://paperpile.com/c/UToEGL/bpaVA+cAg4V). For the remaining dominant-GPSCs the eldest GPS isolate, that assembled into the smallest number of contigs, was selected as a reference for each GPSC. Contigs <500bp were discarded and the remaining contigs were ordered against ATCC 700669 using ABACAS [[49,55]](https://paperpile.com/c/UToEGL/TPk3f+fk0Jw). Ordered and unordered contigs were then compared against ATCC 700669 using BLASTn and ACT used to manually re-order the contigs [[56,57]](https://paperpile.com/c/UToEGL/2xqZc+E1A2e).

Recombination within dominant-GPSCs was quantified using Gubbins from reference mapped alignments for each GPSC [[26]](https://paperpile.com/c/UToEGL/6sk0N). Pairwise SNP distances were calculated for a core gene alignment of the GPS dataset generated via Roary[[27]](https://paperpile.com/c/UToEGL/pBo3O) and recombination-free alignments per dominant-GPSC using the Pairsnp-r R package (https://github.com/gtonkinhill/pairsnp).

Supplementary statistical analysis

Estimates of the number of GPSCs in the true population using total species richness was performed using R version 3.4.1, specpool {vegan}, using chao and chao.se estimates[[28]](https://paperpile.com/c/UToEGL/xcxWg). A genome accumulation plot was generated from a GPSC presence absence matrix for a random sample of n=380 isolates from the 12 countries with >380 samples, using R specaccum {vegan} method = “random” and 100 permutations. The analysis was repeated with 12 independent samples of n=380 from South Africa n=4,615 to demonstrate the effect of deep sampling a single country as opposed to sampling further countries, [https://github.com/rgladstone/GPSCs/blob/master/Gladstone_et_al/Species_richness_GPSC_accumulation.R](https://github.com/rgladstone/GPSCs/blob/master/Gladstone_et_al/species_richness_GPSC_accumulation.R). Simpson’s Diversity index 1-D (SDI) reports no diversity (zero) to unlimited diversity (one) which we calculated for geographical spread and serotype, the latter using only the unperturbed pre-PCV dataset, using R diversity {vegan}<https://github.com/rgladstone/GPSCs/blob/master/Gladstone_et_al/T8-GPSC_summaries.R>.

To test the predictive value of dominant-GPSCs, for antibiogram (unique combinations of resistance to the 5 classes antibiotics assessed here) or serotype, and within each dominant-GPSC the predictive value country for antibiogram or serotype, we used R Anova{Acar} type="III" likelihood ratio test, to compare the R multinom {nnet} logistic regression models to null<https://github.com/rgladstone/GPSCs/blob/master/GPSC_geo_diff_antibiogram_serotype_multinom.R>.

Correction for multiple testing where more than 10 tests were performed used the false discovery rate of 5% R p.adjust {stats}. The pre-PCV dataset representing an unperturbed population was used to identify GPSCs with a significantly higher proportion of penicillin or MDR resistant isolates than the collection overall, and whether the proportion of NVT and VT resistant isolates within GPSCs differed.<https://github.com/rgladstone/GPSCs/blob/master/Gladstone_et_al/T12_T13_GPSC_res_sig.R>

<https://github.com/rgladstone/GPSCs/blob/master/Gladstone_et_al/GPSC_NVT_prop_R.R>

The heritability (*h*^2^) of invasiveness was calculated using the linear-mixed model in pyseer, using a FastTreeMP phylogeny-based distance matrix as the kinship matrix, on a subset of the South African dataset in years 2009-2013 where both carriage and disease isolates were available in children aged <7 years old, excluding those with a known HIV positive status as this host factor influences disease susceptibility (n=1,944, Supplementary T5)[[51,58]](https://paperpile.com/c/UToEGL/NLFcn+YaiID). The explanatory value of serotype for invasiveness was assessed in R via pseudo R^2^ using Nagelkerke {rcompanion} with “leave one out” cross validation using cv {Raster}<https://github.com/rgladstone/GPSCs/blob/master/Gladstone_et_al/pseudoRsq_sero_manifest.R>[[31,59,60]](https://paperpile.com/c/UToEGL/llI8A+w7yzx+WNEnq).

Quantification of invasiveness was performed using odds-ratios (OR) for invasive disease where prevalence in invasive disease was related to their prevalence in carriage, in a meta-analysis of data from South Africa and the USA. For South Africa a random subsample of the heritability dataset (n=1,944) was performed to maintain a constant case to carrier ratio of ~0.57 in each sampling year (n=1,714 Supplementary T6), which includes laboratory-based surveillance of IPD n=625 and cross-sectional colonisation studies; Agincourt n=798, Soweto n=291. Sequence data from children <7 years old from the USA’s pneumococcal active bacterial core surveillance IPD isolates in our GPS dataset, were combined with the published SPARC, and randomly subsampled for a constant case to carrier ratio of ~1.33 (IPD n=456, Carriage n=345) for 2001, 2004, 2007 and 2009, before inclusion in the odds ratio meta-analysis (Supplementary T6). GPSC was inferred from the ST for SPARC 2009 isolates where genomic data was not available [[37,61]](https://paperpile.com/c/UToEGL/2Mh17+eoWMc). The (log) odds ratio used Peto’s method where *a* is the number of invasive isolates of X (serotype, genotype (GPSC or ST) or serotype-genotype), *b* is the number of carriage isolates of X, *c* is the number of non-X invasive isolates, and *d* is the number of non-X carriage isolates in line with previous work by Brueggemann *et al* [7]. This was implemented in R metafor adding 0.5 to each cell in tables with 0 values, with a random effects meta-analysis when >5 isolates in the exposed group were available from both country collections or fixed effects when >5 isolates from only one country [[62]](https://paperpile.com/c/UToEGL/pHo5A). The Cochran’s Q-test was used to detect heterogeneity within the estimated OR. Differences in proportions of IPD cases to carriage were performed between the most and least invasive genotypes within the country they were predominantly observed as the case carrier ratios and temporal spread differed between the two collections using Fisher’s exact test.<https://github.com/rgladstone/GPSCs/blob/master/Gladstone_et_al/OR_meta_ZAUS_GPSC_ST.R>

References:

[47] [Epping L, Hunt M, van Tonder AJ, Gladstone RA, The Global Pneumococcal Sequencing consortium, Bentley SD, et al. SeroBA: rapid high-throughput serotyping of Streptococcus pneumoniae from whole genome sequence data. bioRxiv 2017:179465. doi:](http://paperpile.com/b/UToEGL/iAHS)[10.1101/179465](http://dx.doi.org/10.1101/179465)[.](http://paperpile.com/b/UToEGL/iAHS)

[48] [Hunt M, Mather AE, Sánchez-Busó L, Page AJ, Parkhill J, Keane JA, et al. ARIBA: rapid antimicrobial resistance genotyping directly from sequencing reads. Microb Genom 2017;3:e000131.](http://paperpile.com/b/UToEGL/MlPTA)

[49] [Croucher NJ, Walker D, Romero P, Lennard N, Paterson GK, Bason NC, et al. Role of Conjugative Elements in the Evolution of the Multidrug-Resistant Pandemic Clone Streptococcus pneumoniaeSpain23F ST81. J Bacteriol 2009;191:1480–9.](http://paperpile.com/b/UToEGL/TPk3f)

[50] [Page AJ, Taylor B, Delaney AJ, Soares J, Seemann T, Keane JA, et al. SNP-sites: rapid efficient extraction of SNPs from multi-FASTA alignments. Microb Genom 2016;2:e000056.](http://paperpile.com/b/UToEGL/DyHIu)

[51] [Price MN, Dehal PS, Arkin AP. FastTree 2 – Approximately Maximum-Likelihood Trees for Large Alignments. PLoS One 2010;5:e9490.](http://paperpile.com/b/UToEGL/NLFcn)

[52] [PacBio. SMRT-Analysis: A software suite for analyzing single molecule, real-time DNA sequencing data 2016.](http://paperpile.com/b/UToEGL/DyftH) <https://github.com/PacificBiosciences/SMRT-Analysis>[.](http://paperpile.com/b/UToEGL/DyftH)

[53] [Walker BJ, Abeel T, Shea T, Priest M, Abouelliel A, Sakthikumar S, et al. Pilon: an integrated tool for comprehensive microbial variant detection and genome assembly improvement. PLoS One 2014;9:e112963.](http://paperpile.com/b/UToEGL/bpaVA)

[54] [Hunt M, Silva ND, Otto TD, Parkhill J, Keane JA, Harris SR. Circlator: automated circularization of genome assemblies using long sequencing reads. Genome Biol 2015;16:294.](http://paperpile.com/b/UToEGL/cAg4V)

[55] [Wellcome Trust Sanger Institute. ABACAS 2015.](http://paperpile.com/b/UToEGL/fk0Jw) <http://abacas.sourceforge.net/>[.](http://paperpile.com/b/UToEGL/fk0Jw)

[56] [Altschul SF, Gish W, Miller W, Myers EW, Lipman DJ. Basic local alignment search tool. J Mol Biol 1990;215:403–10.](http://paperpile.com/b/UToEGL/2xqZc)

[57] [Carver TJ, Rutherford KM, Berriman M, Rajandream M-A, Barrell BG, Parkhill J. ACT: the Artemis comparison tool. Bioinformatics 2005;21:3422–3.](http://paperpile.com/b/UToEGL/E1A2e)

[58] [Lees J, Galardini M, Bentley SD, Weiser JN, Corander J. pyseer: a comprehensive tool for microbial pangenome-wide association studies. bioRxiv 2018:266312. doi:](http://paperpile.com/b/UToEGL/YaiID)[10.1101/266312](http://dx.doi.org/10.1101/266312)[.](http://paperpile.com/b/UToEGL/YaiID)

[59] [Mangiafico S. rcompanion: Functions to support extension education program evaluation. R Package Version 1 5 0 The Comprehensive R Archive Network 2017.](http://paperpile.com/b/UToEGL/llI8A)

[60] [Hijmans RJ, van Etten J. raster: Geographic data analysis and modeling. R Package Version 2014;2.](http://paperpile.com/b/UToEGL/w7yzx)

[61] [Hanage WP, Bishop CJ, Lee GM, Lipsitch M, Stevenson A, Rifas-Shiman SL, et al. Clonal replacement among 19A Streptococcus pneumoniae in Massachusetts, prior to 13 valent conjugate vaccination. Vaccine 2011;29:8877–81.](http://paperpile.com/b/UToEGL/2Mh17)

[62] [Viechtbauer W, Others. Conducting meta-analyses in R with the metafor package. J Stat Softw 2010;36:1–48.](http://paperpile.com/b/UToEGL/pHo5A)
