# Supplementary material for: International genomic definition of pneumococcal lineages, to contextualise disease, antibiotic resistance and vaccine impact
Source: eBioMedicine. 2019 Apr 16;43:338–46. doi: 10.1016/j.ebiom.2019.04.021 (PMC6557916; doi:10.1016/j.ebiom.2019.04.021)
Supplement: Supplementary file 4 — Supplementary Figures [file mmc4.docx]

Figure S1

Genomic distances that define the between and within pneumococcal lineages threshold


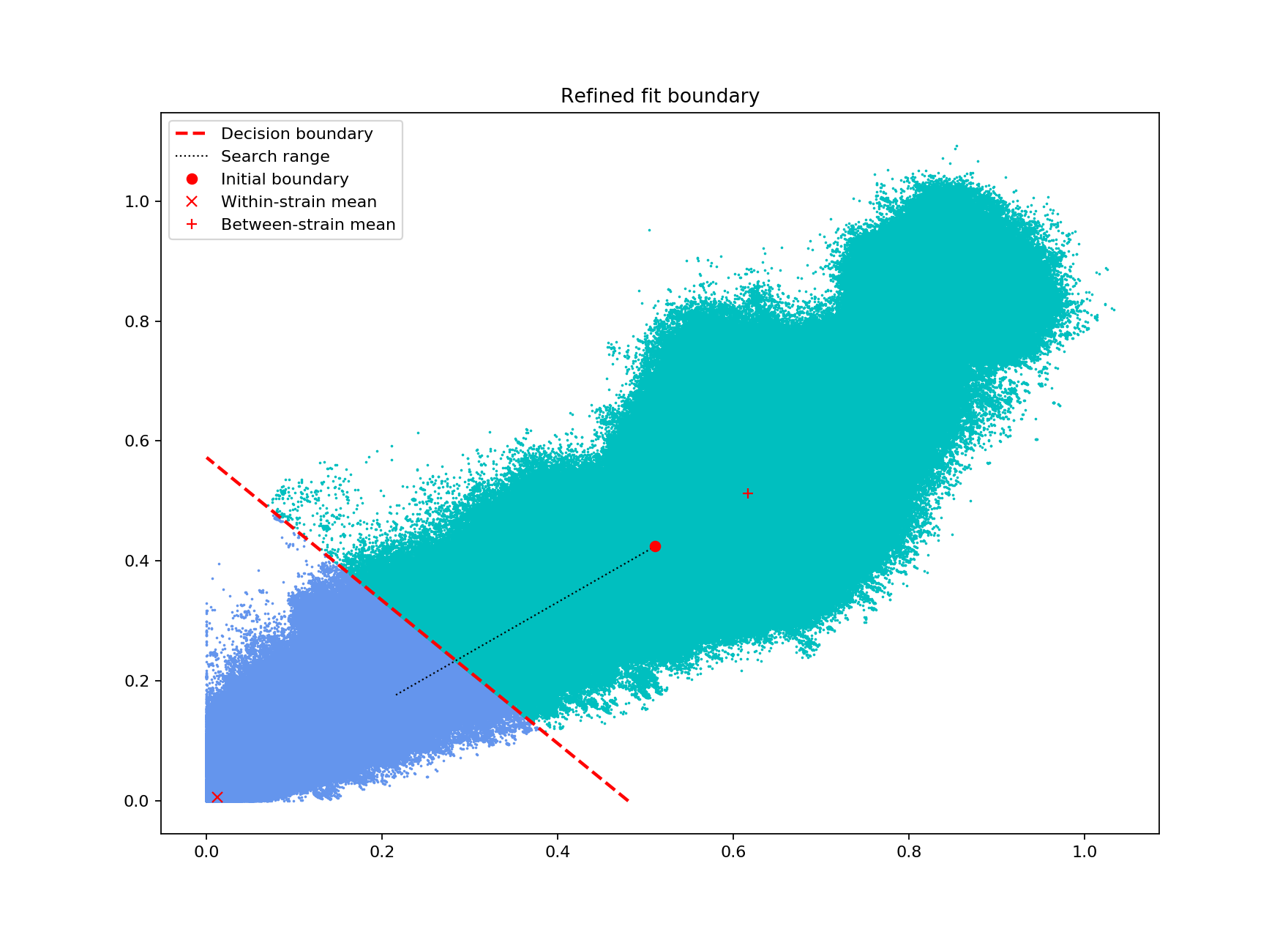


Jaccard distance

Core genome nucleotide divergence

PopPUNK was used to determine core and accessory distances for n=20,027 isolates, calculated by shared DNA k-mers with lengths between 13 and 29 bases to optimising the decision boundary for within GPSC (blue) or between GPSCs (green) on the combined distances (red dashed line).

Figure S2

Geographical sampling of Global Pneumococcal Sequencing (GPS) collection n=13,454 by carriage and disease


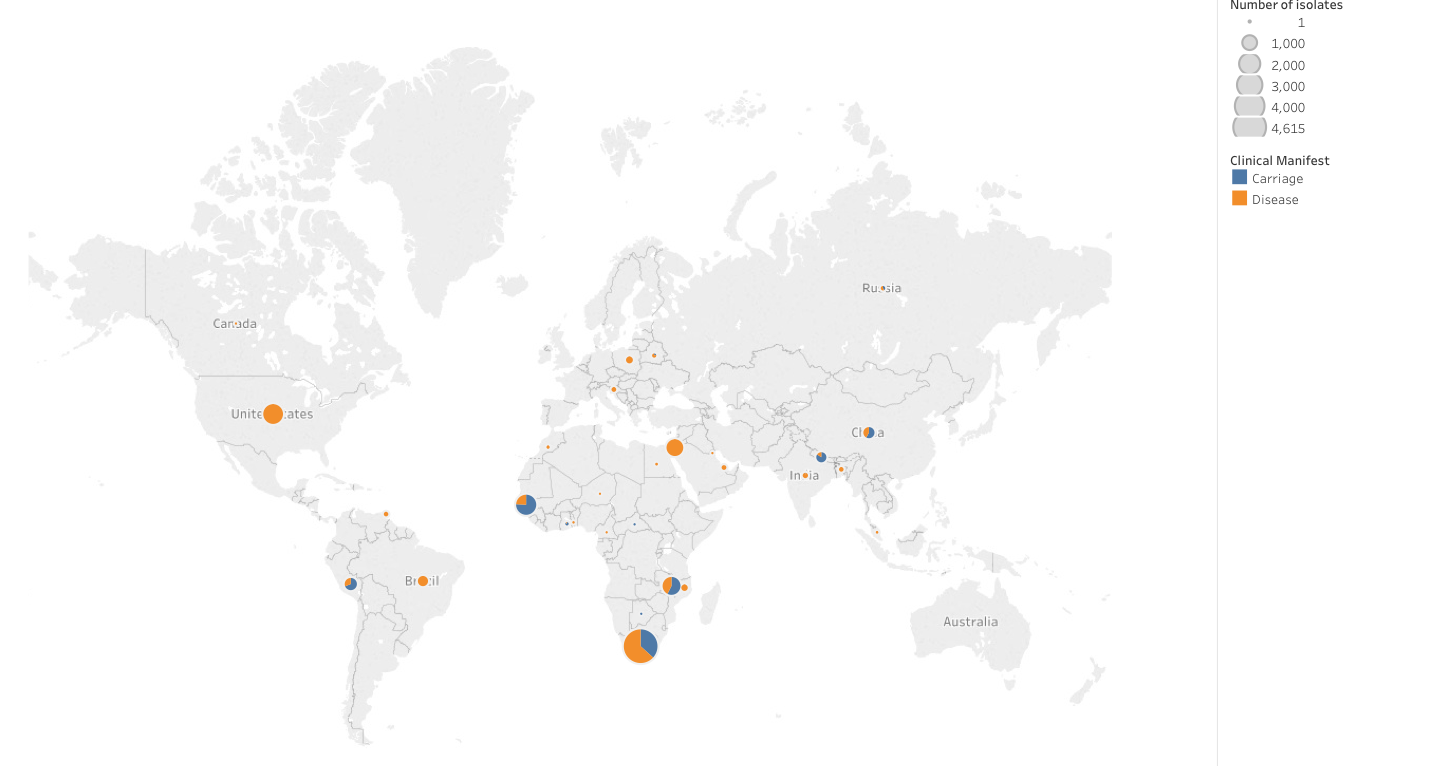


The proportion of carriage (blue) and disease (orange) isolates from the GPS dataset n=13,454 by geographical location, where the size of the pie is scaled to the number of isolates (see key).

Figure S3. Global Pneumococcal Sequence Clusters (GPSCs)
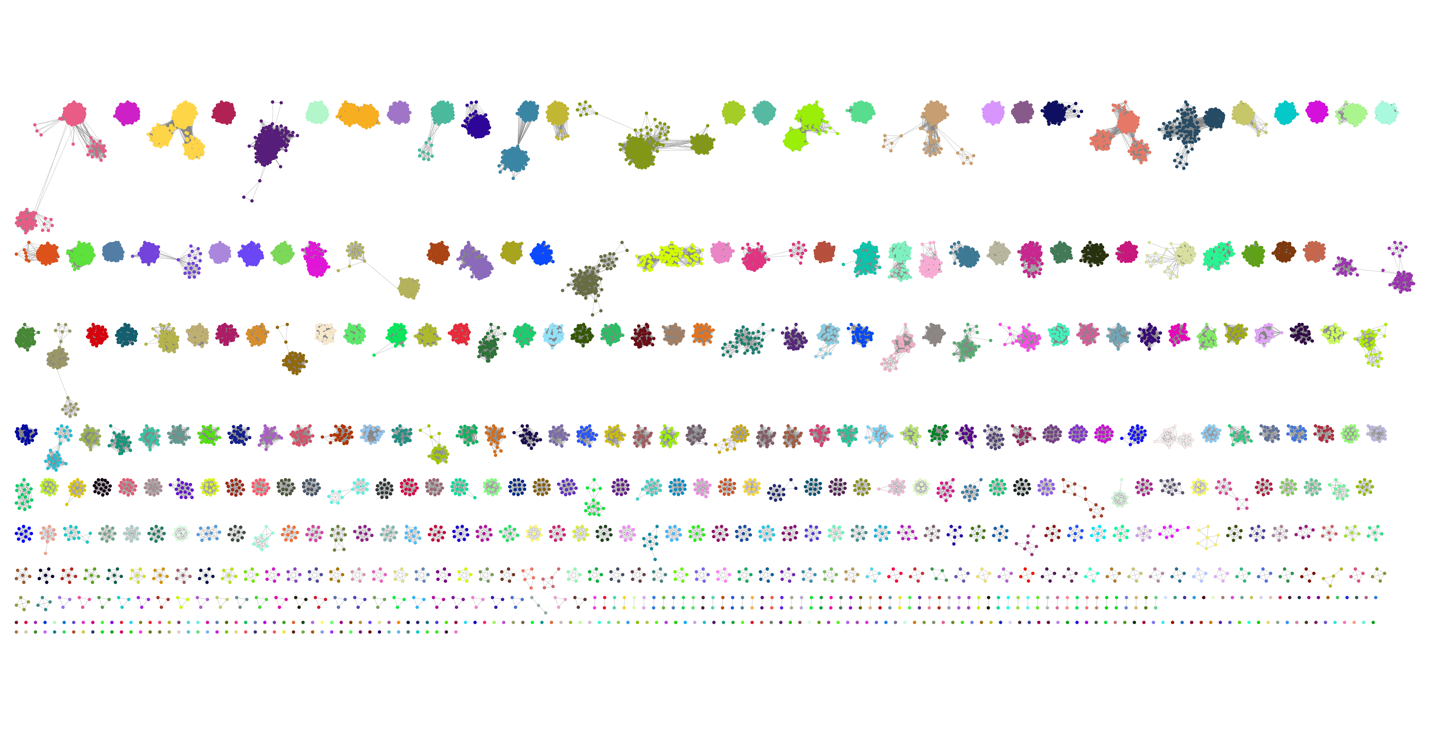


PopPUNK clustered 20,027 isolates into the 621 clusters displayed here. Each dot in this network represents an individual isolate. Isolates are coloured according to their assigned clusters, each cluster of linked isolates depicts the ranked GPSCs from GPSC1 (n=885), through to GPSC621 (n=1) from left to right.

Figure S4

GPSCs ranked by the number of pneumococcal isolates representing them


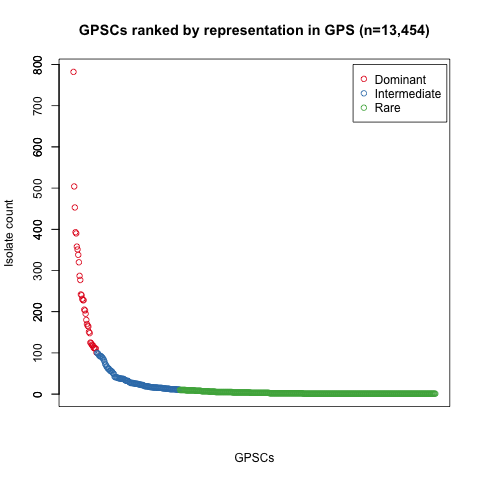


Each circle represents a GPSC and the number of isolates representing it, for disease and carriage combined (n=13,454). GPSCs were classified into dominant-GPSCs if n>100, which accounted for 62% of our collection n=13,454. Rare GPSCs were defined as those represented by <10 isolates, with the remaining GPSCs classified as intermediate.

Figure S5: Accumulation of observed GPSCs

A) B)


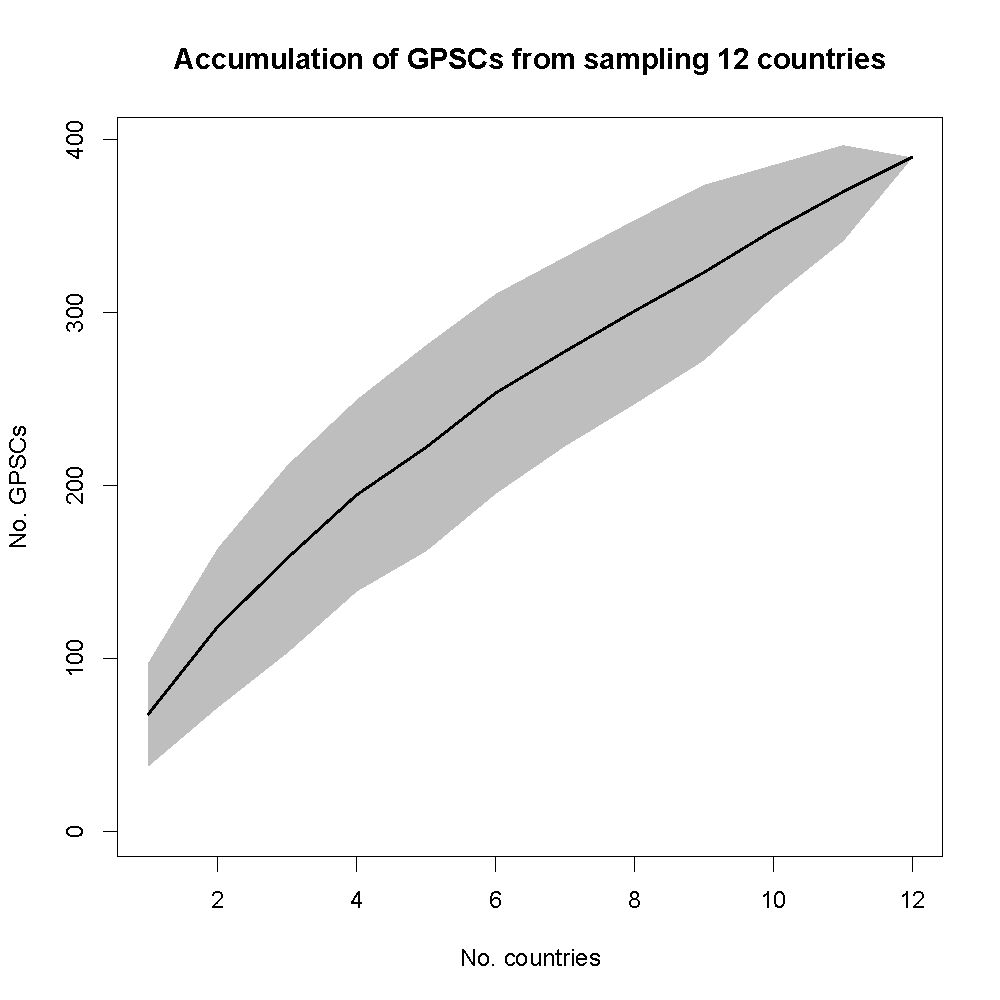

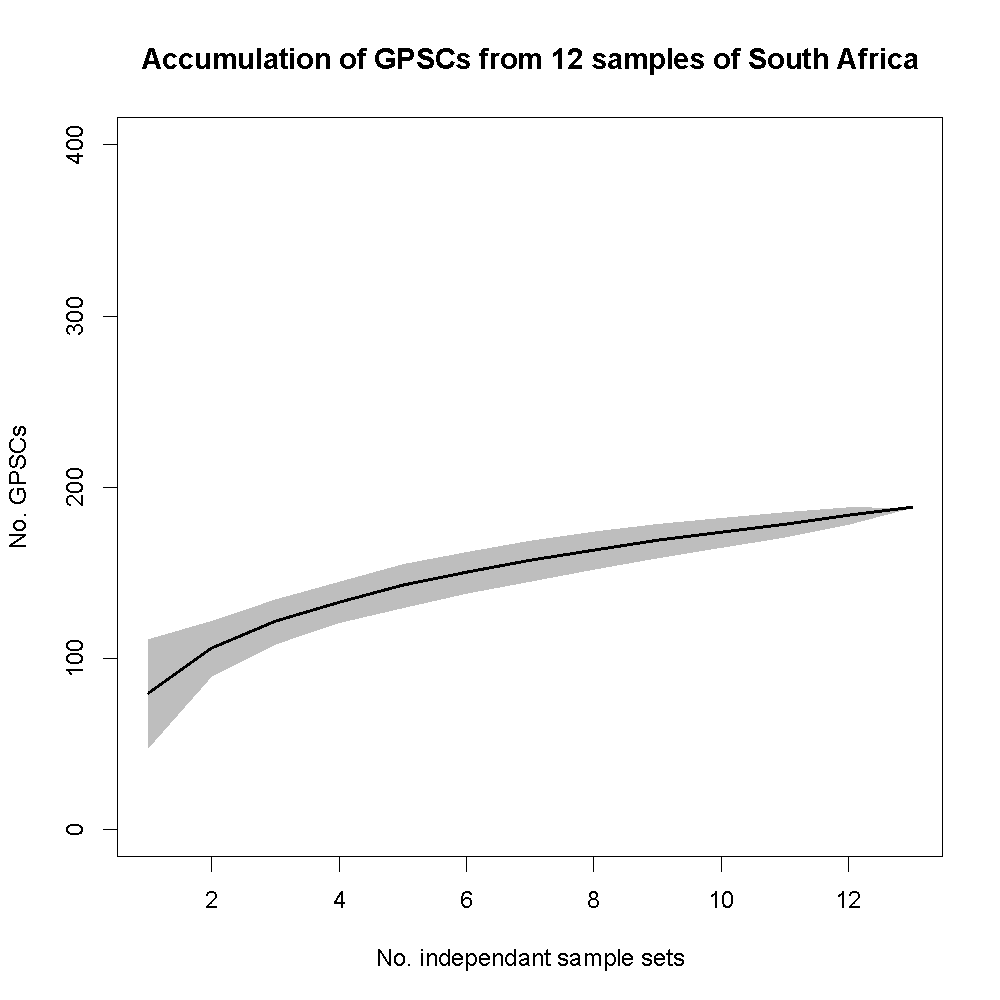


Accumulation of observed GPSCs in 100 permutations adding datasets in a random order where the datasets are A) extended geographical sampling of n=380 isolates from 12 locations or A) twelve independent samples of n=380 from a single country (South Africa). Black line represents estimated number of GPSCs, grey area represents confidence intervals from standard deviation.

Figure S6. Pneumococcal MLST clonal complexes on a core genome phylogeny of Global Pneumococcal Sequence Cluster representatives


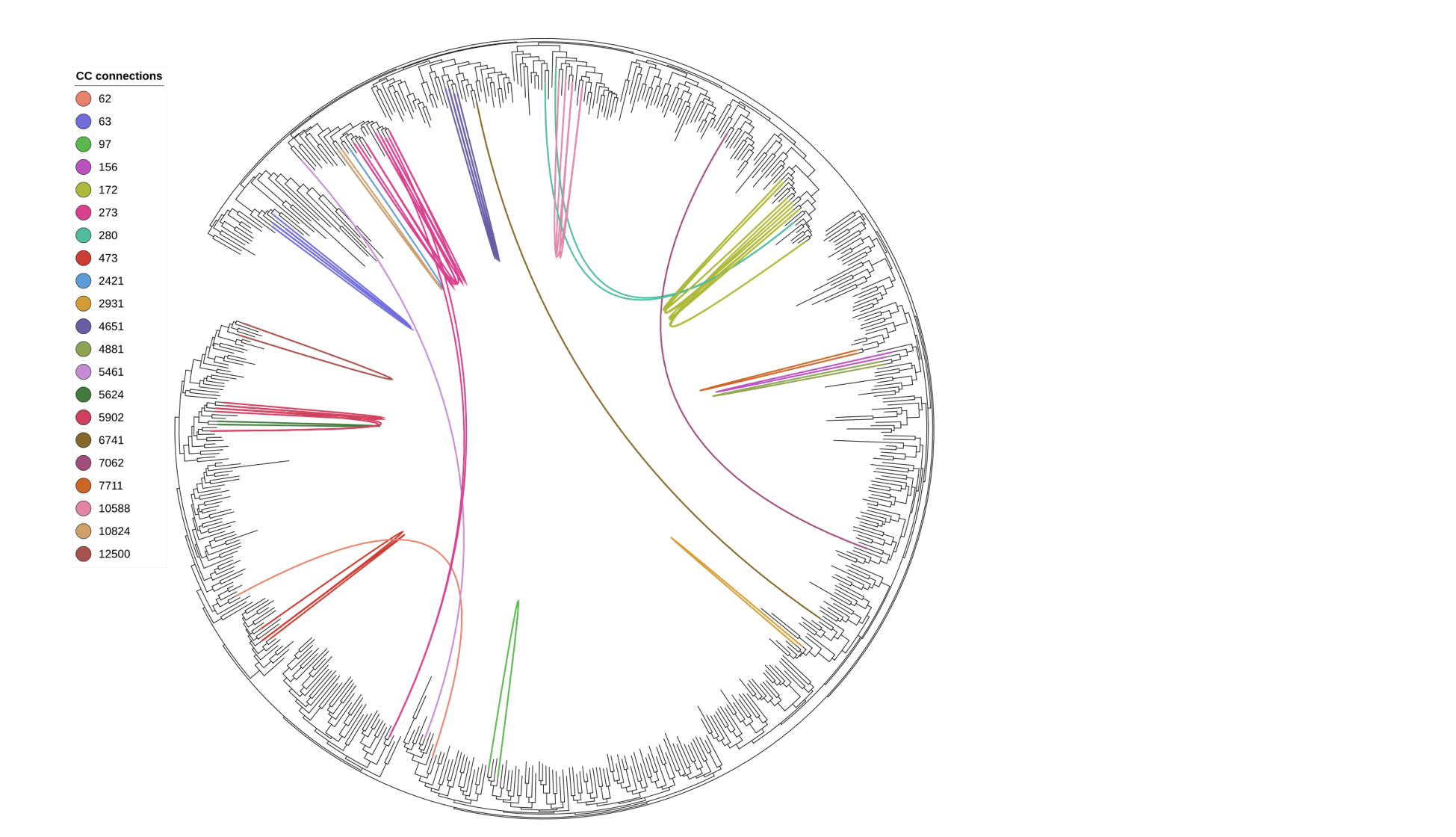
 Each taxon in this core genome phylogeny is a single representative of each of the 621 Global Pneumococcal Sequence Clusters (GPSCs). Lines link multiple GPSCs that have isolates assigned to the same clonal complex (CC), and are coloured by CC. The majority of GPSCs entirely encompass a CC, with an average 2.6 CCs per GPSC. When CCs span multiple GPSCs, the majority (15/21) span GPSCs that are closely related on the phylogeny. The remaining 6 CCs span GPSCs that have no close phylogenetic relationship. Interactive Figure S6 available at <https://itol.embl.de/tree/193622058175841530027280>

Figure S7. Pneumococcal resistance frequency by antibiotic class


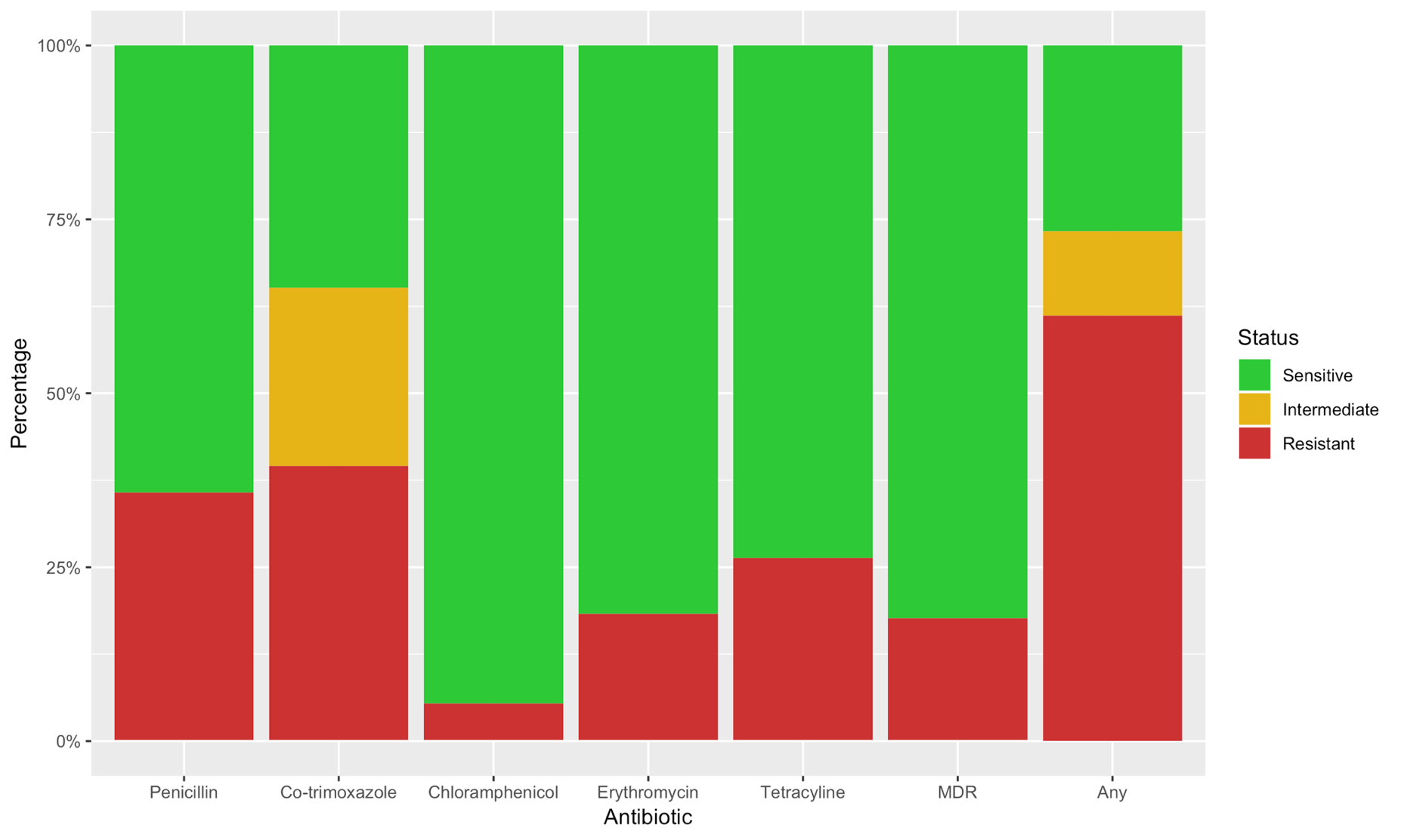


Stacked bar chart showing the percentage of isolates in the GPS dataset predicted as being resistant/intermediate/sensitive to each antibiotic class, multidrug resistant (>=3 of the antibiotic classes) or resistance to any of these antibiotic classes. N.B. intermediate susceptibility cannot be predicted for antibiotic classes where gene presence or absence determines resistance or susceptibility respectively.

Figure S8. Differences in pneumococcal invasiveness within serotypes due to genotype.


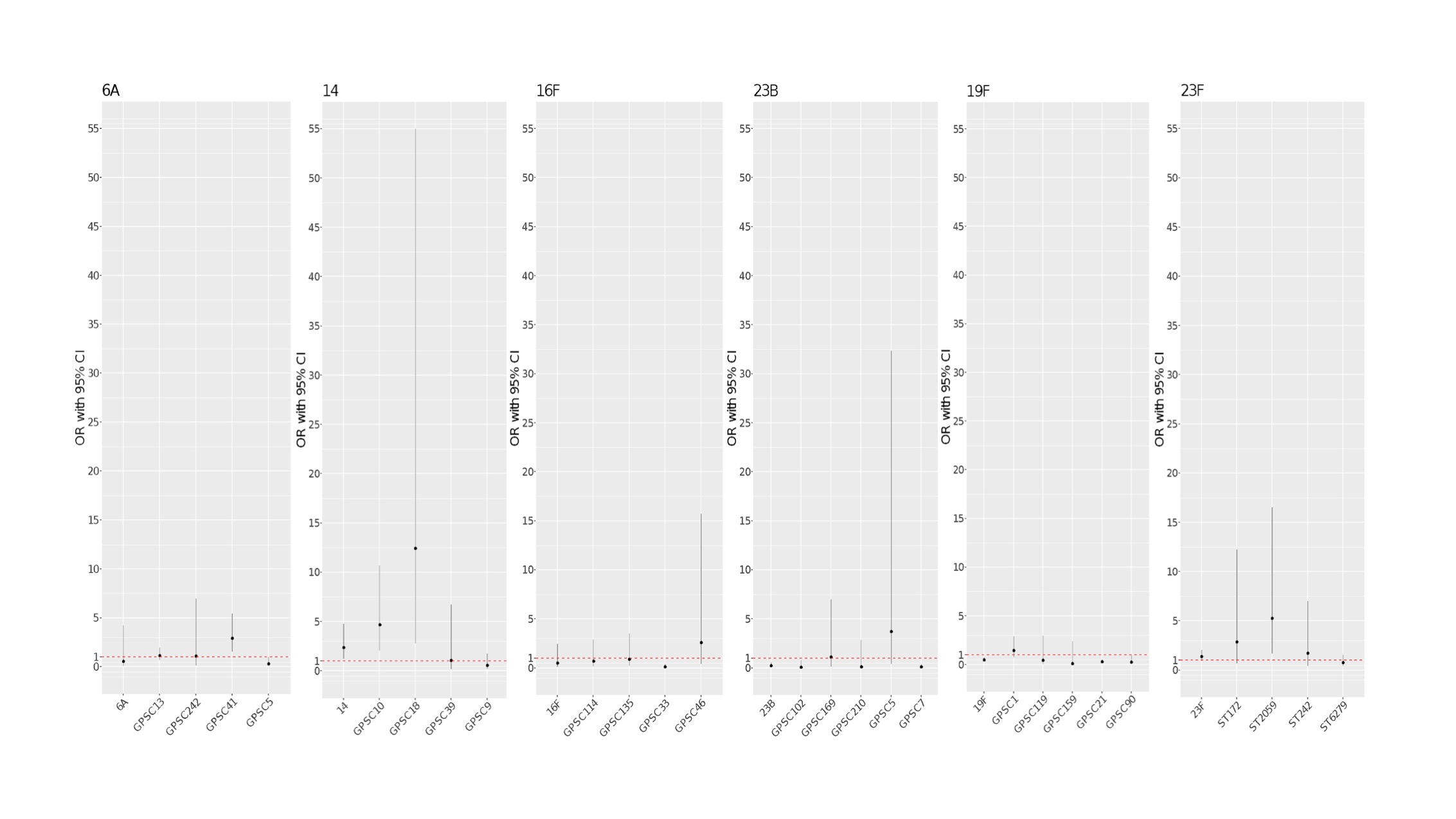
Log odds ratios (OR) for invasiveness from meta-analysis of carriage and disease isolates from South Africa (n=1,944, case:carrier 0.57) and USA (n=801, case:carrier 1.33), are plotted with 95% CI for each genotype within a serotype background if one genotype had a non-overlapping CI indicating it is more invasive than the other genotype. OR threshold in marked with a dashed red, where >1 invasive.
